# Bacteria-Derived Cellulose Membranes Modified with Graphene Oxide-Silver Nanoparticles for Accelerating Wound Healing

**DOI:** 10.1021/acsabm.4c00650

**Published:** 2024-08-02

**Authors:** Erika Patrícia Chagas Gomes Luz, Thamyres Freire da Silva, Lidyane Souto Maciel Marques, Alexandre Andrade, Marcos Vinicius V Lorevice, Fabia Karine Andrade, Liu Yang, Antonio Gomes de Souza Filho, Andreia F. Faria, Rodrigo Silveira Vieira

**Affiliations:** † Department of Chemical Engineering, Federal University of Ceará (UFC), Fortaleza, Ceará 60455-760, Brazil; ‡ Department of Pathology and Forensic Medicine, Federal University of Ceará (UFC), Fortaleza, Ceará 60430-160, Brazil; § Department of Physics, Federal University of Ceará (UFC), Bloco 922, Fortaleza, Ceará 60455-760, Brazil; ∥ Department of Environmental Engineering Sciences, University of Florida, Gainesville, Florida 32611-6540, United States; ⊥ Brazilian Nanotechnology National Laboratory (LNNano), Brazilian Center for Research in Energy and Materials (CNPEM), Campinas, São Paulo 13083-970, Brazil

**Keywords:** bacterial cellulose, graphene oxide, silver nanoparticles, wound dressings, skin wounds, wound healing, antimicrobial agents

## Abstract

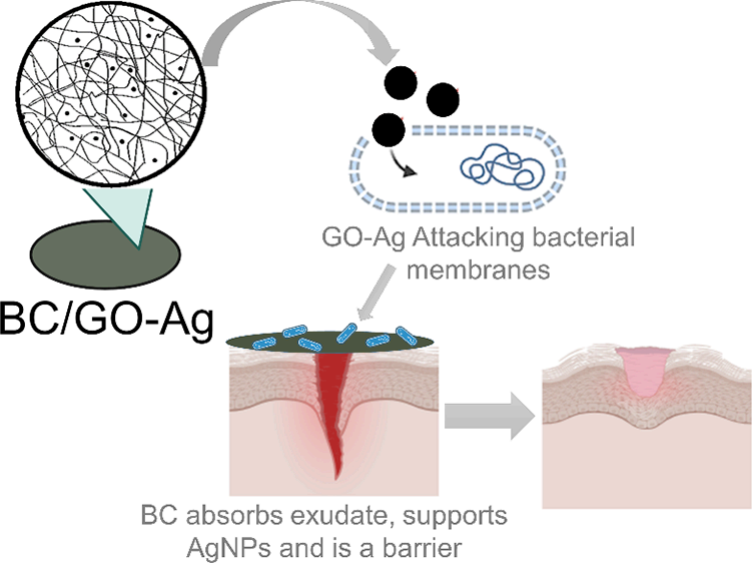

This study reports on the modification of bacterial cellulose (BC) membranes produced by static fermentation of *Komagataeibacter xylinus* bacterial strains with graphene oxide-silver nanoparticles (GO-Ag) to yield skin wound dressings with improved antibacterial properties. The GO-Ag sheets were synthesized through chemical reduction with sodium citrate and were utilized to functionalize the BC membranes (BC/GO-Ag). The BC/GO-Ag composites were characterized to determine their surface charge, morphology, exudate absorption, antimicrobial activity, and cytotoxicity by using fibroblast cells. The antimicrobial activity of the wound dressings was assessed against *Staphylococcus aureus*, *Escherichia coli*, and *Pseudomonas aeruginosa*. The results indicate that the BC/GO-Ag dressings can inhibit ∼70% of *E. coli* cells. Our findings also revealed that the porous BC/GO-Ag antimicrobial dressings can efficiently retain 94% of exudate absorption after exposure to simulated body fluid (SBF) for 24 h. These results suggest that the dressings could absorb excess exudate from the wound during clinical application, maintaining adequate moisture, and promoting the proliferation of epithelial cells. The BC/GO-Ag hybrid materials exhibited excellent mechanical flexibility and low cytotoxicity to fibroblast cells, making excellent wound dressings able to control bacterial infectious processes and promote the fast healing of dermal lesions.

## Introduction

Skin wounds have become an essential medical issue within the healthcare system due to the physical and financial burden they impose on patients and hospitals.^1−3^ Efficient treatments for skin lesions should be performed to avoid complications during the healing process and prevent the patient’s condition from progressing into chronic wounds. Chronic skin wounds can lead to high levels of inflammation and infections due to difficulties repairing the epithelial and granular tissues.^4,5^ When not adequately cared for, severe burns, pressure ulcers (decubitus), and venous ulcers can transition into chronic wounds.^6−8^ Additionally, comorbidities associated with the patient, such as age, diabetes, heart disease, and obesity, can complicate wound healing.^9,10^

A small skin lesion usually recovers within days through an arrangement of the organism to promote cell migration and maintain appropriate levels of inflammation, innervation, and angiogenesis. However, more severe wounds may take weeks to heal and leave a visible scar. Chronic wounds, on the other hand, cannot follow this natural physiological process, leading to an extended healing period from months to years, making it a major therapeutic challenge worldwide.^6,11^ This is due to the growing number of cases, high treatment costs, clinical limitations of systemic drugs, and negative impact on the life quality of patients.^12,13^ To address this issue, researchers have developed wound dressings that promote healing. Antimicrobial dressings, for example, can minimize the effects of microorganism contamination, such as pathogenic bacteria and fungi, and help remove dead tissue, exudate, and pus, which can improve the healing rate without compromising the vital functions of the human body.^14^

Researchers in academia and industry are working to develop advanced dressings that can promote faster healing of skin wounds while preventing infection.^15,16^ A previous study has shown that combining bacterial cellulose and alginate loaded with papain can create wound dressings that are anti-inflammatory and have high porosity, fluid absorption, biocompatibility, low cytotoxicity, and controlled and stabilized papain release.^17,18^ In another study, antimicrobial dressings made from seaweed, poly(vinyl alcohol), and polyvinylpyrrolidone were performed similarly to Acticoat silver dressing, inactivating 70 to 90% of pathogens commonly found in wounds.^19^ Antimicrobial and anti-inflammatory properties are essential for wound dressings as they can help promote skin regeneration and healing after injury.

Lately, numerous studies have focused on experimenting with various combinations of materials in order to create biomaterials with unique and enhanced properties. The medical field, particularly in the area of epithelial regeneration, has been striving to develop biomaterials by integrating active ingredients that can expedite tissue regeneration. Many of these active ingredients possess antimicrobial and antioxidant properties, as demonstrated in a study by Riaz et al. In this study, a hydrogel was developed using a combination of l-phenylalanine, agarose, and gallic acid, which was capable of eliminating free radicals and showed antibacterial effects against both Gram-positive and Gram-negative bacteria.^20^ Additionally, there are dressings created through electrospinning that are designed for tissue repair and enable the simultaneous administration of medications to prevent adhesions, reduce inflammation, and promote healing.^21^ Furthermore, in studies involving electrospun dressings, this process also aids in the ability to manipulate product formats and modulate drug release.^22^

Different substances are incorporated into cellulose and derivatives to contribute to the antimicrobial action of dressings, such as natural oils,^23−25^ metal ions,^26−29^ or a mixture with antimicrobial polymers.^30−32^ Nanomaterials, particularly graphene oxide (GO) and its composites, have intrinsic properties that make them suitable for biomedical applications. These materials exhibit high specific surface area, biocompatibility, and hydrophilicity. Furthermore, GO sheets have been found to exhibit *in vitro* antibacterial activity and noncytotoxicity at relatively low concentrations.^33−37^ Additionally, hydroxyl, epoxy, and carboxylic functional groups on the GO sheets can be leveraged as nucleation sites to anchor silver nanoparticles (AgNPs), enhancing their antibacterial properties and expanding their bactericidal spectrum. The advantage of using silver anchored to GO is that the Ag^+^ ions are released more slowly,^38^ helping to prolong the antimicrobial effect while maintaining a therapeutically effective maintenance dose.

Due to the demand for technologically advanced dressings, this research proposes the development of an advanced antimicrobial dressing made with bacterial cellulose (BC) and graphene oxide (GO) modified with silver nanoparticles (AgNPs). The GO-Ag composite material was synthesized using silver nitrate and sodium citrate as the silver precursor and reducing agent, respectively. GO-Ag was characterized through Raman spectroscopy, TEM, and X-ray spectroscopy to verify the attachment of AgNPs to the surface of GO sheets. The BC was biologically produced through static fermentation, purified, and used as a polymeric base for the adsorption of GO-Ag nanosheets, creating the functionalized BC/GO-Ag dressing. The BC/GO-Ag dressings were tested against their ability to absorb simulated body fluid (SBF) and release silver ions. The dressing was also tested for its antimicrobial activity and cytotoxicity in fibroblast cells (L-929) to demonstrate its feasibility in treating human skin wounds.

## Experimental Section

### Chemicals and Materials

Single-layer GO (Cheap Tubes), tribasic sodium citrate (Na_3_C_6_H_5_O_7_, Cromoline), silver nitrate (AgNO_3_, ≥98% Synth), potassium carbonate (K_2_CO_3_, Dynamic), agar–agar (bacteriological, Dynamic), sodium chloride (NaCl, Neon), Mueller–Hilton broth (M391, HiMedia), fetal bovine serum (F0804, Sigma-Aldrich), Dulbecco’s Modified Eagle's Medium–high glycose (D5648, Sigma-Aldrich), penicillin–streptomycin (P4333, Sigma-Aldrich), resazurin (R7017, Sigma-Adrich), HS broth: citric acid (C_6_H_8_O_7_, Neon), yeast extract (NCM0218A-HX0161-00055, Neogen), d-(+)-glucose (C_6_H_12_O_6_, Neon), dibasic sodium phosphate (Na_2_HPO_4_, Dynamics), and peptone (bacteriological, Kasvi) were the chemicals and materials used for this study.

### Production and Purification of BC

BC was obtained from *Komagataeibacter hansenii* (ATCC 53582) and cultivated under static conditions. The detailed protocol for the process has been described extensively in a published paper.^39^ The cell mass from the stock culture containing the slant agar was transferred onto an HS medium plate to initiate the process. The strain was activated by streaking and incubation at 30 °C for 48 h. The resulting mass was transferred to Scott flasks containing 100 mL of HS broth and incubated at 30 °C for 48 h. The production was terminated by transferring 10% (v/v) of the broth into new HS broth in Scott flasks (86 mm diameter), followed by incubation at 30 °C for 5 days. The BC membrane, an extracellular metabolite, was formed on the surface of the broth. Using a spatula, the BC membrane was removed from the fermenting medium, washed with distilled water (DW), and treated twice with 0.3 mol L^–1^ of potassium carbonate (K_2_CO_3_) at 80 °C for 1 h. Afterward, the BC membranes were rinsed with water at room temperature until reaching a neutral pH. Details of this protocol can be found in our previous publications.^39−42^

### GO-Ag Synthesis and Characterization

GO-Ag nanoparticles were synthesized in a lower concentration of graphene oxide (GO1), citrate, and silver (GO-Ag1) and another containing two times higher concentrations of graphene oxide (GO2), citrate, and silver (GO-Ag2). The antibacterial performance of GO1 and GO2 was evaluated using a methodology described.^43,44^ First, GO was dispersed in DW (312.5 and 625 μg mL^–1^) and then sonicated using a probe sonicator (Hielscher, UP100H) for 30 min and 50% frequency for 0.5 cycles in an ice bath. Then, AgNO_3_ (267 and 534 μg mL^–1^) was added to the GO suspension and left under stirring for 30 min in an ice bath away from light. These mixtures were taken to a probe sonicator for 5 min to promote the interaction between GO sheets and silver ions. Then, these mixtures were placed into a reflux system at a boiling temperature (∼130 °C). As soon as the GO-silver suspension reached the boiling point, sodium citrate (250 and 500 μg mL^–1^) was added by constant dripping. The systems were then kept in contact for 50 min at 130 °C.

The structural morphology and chemical elemental analysis of GO-Ag1 and GO-Ag2 were determined by a transmission electron microscope (TEM, FEI Titan Cubed Themis, Thermo Fischer) coupled with an energy-dispersive X-ray spectroscopy detector operating at 300 kV equipped with a Ceta camera 4k × 4k. The particle size distribution of GO1, GO-Ag1, GO2, and GO-Ag2 was determined by dynamic light scattering (DLS) using a Zetasizer (ZEN 3600, Malvern) with a red-light beam and wavelength of 633 nm. The experiment was conducted at 25 °C using GO1, GO-Ag1, GO2, and GO-Ag2 dissolved in water. The structural properties of GO1, GO-Ag1, GO2, and GO-Ag2 were analyzed using Raman spectroscopy, WITec alpha300, with an Andor optical system and coherent anti-Stokes scattering (CARS). In addition, XRD patterns of the samples were recorded using a PANalytical X’Pert PRO (Netherlands) diffractometer with a scanning scope of 10–60°, operating at 40 kV and scanning speed of 0.13° min^–1^, using Co Kα radiation (λ = 1.789).

### BC and GO-Ag Combine to Produce BC/GO-Ag Wound Dressings

**Figure 1:**
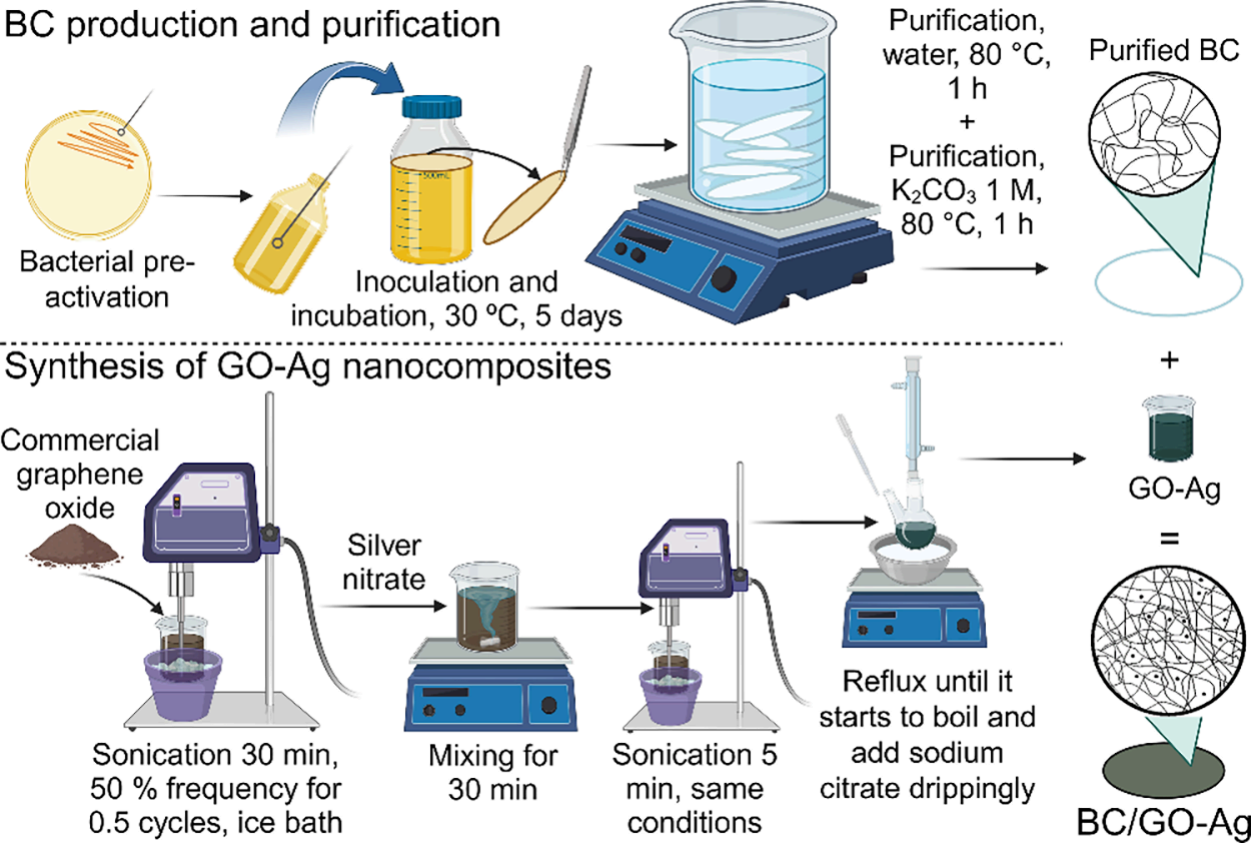
Schematic production of the BC/GO-Ag dressing. *Komagataeibacter xylinus* (ATCC 53582) is cultivated for 5 days at 30 °C in HS medium to produce the BC membrane, and GO-Ag nanocomposites are synthesized by applying sodium citrate as a reducing agent. The BC/GO-Ag dressing is prepared by contacting the BC membranes with a GO-Ag suspension for 3 h under agitation at 25 °C.

The BC/GO-Ag dressings were produced by soaking the BC dressings (an equivalent of 0.013 g of dry mass) in GO and GO-Ag suspensions (the ratio of GO and Ag was 1.21) at the two different concentrations. The BC membranes and graphene solutions were kept in contact at 25 °C in a water bath for 24 h under agitation. Then, the dressings were removed, rinsed with DW, and the solution was autoclaved at 121 °C for 15 min. Figure 1 depicts the schematic production of BC, GO-Ag, and BC/GO-Ag. The BC/GO1, BC/GO-Ag1, BC/GO2, and BC/GO-Ag2 dressings were characterized using a Raman spectrometer (alpha300, WITec, Germany) equipped with an optical microscopy system and CARS to determine the characteristic presence of the D and G bands attributed to GO. The morphology and chemical elemental analyses of BC/GO1, BC/GO-Ag1, BC/GO2, and BC/GO-Ag2 were determined by SEM (Quanta, 450 FEG FEI, PAIS) coupled with energy-dispersive X-ray spectroscopy (JEM-1011, JEOL), respectively.

The swelling degree was determined using the method described by Liu and collaborators.^45^ The BC/GO1, BC/GO-Ag1, BC/GO2, and BC/GO-Ag2 dressings (16 mm diameter) were immersed in water, and after each immersion, the excess water was removed using filter paper (Quanty; 8 μm) and weighed at intervals of 0, 0.017, 0.116, 0.200, 0.367, and 0.5 h at 25 °C. The degree of swelling was expressed as the percentage of mass gain compared to the initial mass, according to eq 1.
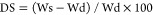
1where DS is the degree of swelling, Ws is the mass of the sample after immersion, and Wd is the mass of the dry sample before immersion. All measurements were carried out in triplicate.

The exudate absorption capacity of the dressings was determined by the gravimetric method through the degree of swelling in SBF (pH 7.4 and composition: 142.0 mM Na^+^, 5.0 mM K^+^, 2.5 mM Ca^2+^, 1.5 mM Mg^2+^, 148.8 mM Cl^–^, 4.2 mM HCO_3_^–^, 1.0 mM HPO_4_^–2^, 0.5 mM SO_4_^–2^).

The BC/GO1, BC/GO-Ag1, BC/GO2, and BC/GO-Ag2 dressings were previously dried (lyophilized), cut into pieces 16 mm in diameter, and weighed to determine their initial mass (*m*_initial_). Then, the membranes were immersed in 10 mL of the simulated body fluid for 24, 48, and 72 h at 37 °C. After exposure, the excess liquid was removed with filter paper. Finally, the samples were weighed again on an analytical balance to determine the final wet masses (*m*_wet_). The absorption capacity (*Q*) was calculated by using eq 2, and the value was expressed as a percentage.
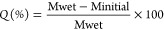
2

The release of silver ions (Ag^+^) from the BC/GO-Ag1 and BC/GO-Ag2 samples was quantified by atomic absorption spectrometry (AAS). BC/GO-Ag1 and BC/GO-Ag2 samples (50.27 cm^2^) were placed in 10 mL of DW and 37 °C under agitation, and at each experimental time, the entire solution was removed and replaced by new purified water at a defined period (48 h).

### Antibacterial Activity of BC, BC/GO, and BC/GO-Ag Dressings

The antimicrobial activity was determined via an indirect method, using the extracts from the materials. The BC/GO1, BC/GO-Ag1, BC/GO2, and BC/GO-Ag2 dressings were kept in contact with PBS incubated at 37 °C for 24 h, respecting an established proportion of 1 mL of PBS per cm^2^ of the dressings. The samples were tested against the Gram-positive bacterium *Staphylococcus aureus* (ATCC 25923) and the Gram-negative bacteria *Pseudomonas aeruginosa* (ATCC 25619) and *Escherichia coli* (ATCC 8739), as described by Oliveira et al.^46^ The bacteria were placed in fresh Mueller–Hinton media and incubated for 24 h at 37 °C before the assay. Briefly, a 50 μL aliquot of Mueller–Hinton culture medium with the tested bacteria (O.D. 0.1 at 600 nm) was incubated in the dark at 37 °C with 50 μL of the supernatant originating from the contact BC/GO1, BC/GO-Ag1, BC/GO2, and BC/GO-Ag2 with the PBS solution. After 24 h, the bacterial growth was evaluated by absorbance at 600 nm by using an automated microplate reader (Epoch, BioTek Instruments Inc., USA). The commercial dressings Aquacel Ag Extra and Mepilex Ag+ Molnlycke were used as positive controls. The negative control was bacteria exposed to a Mueller–Hinton culture medium. All assays were performed in triplicate, and the results were expressed as mean percentages with standard deviations.

### *In Vitro* Cytotoxicity Assay against Mouse Fibroblasts L-929

The cytotoxicity assay was performed and obtained according to ISO10993-12 and ISO10993-5.^47,48^ Initially, the BC/GO1, BC/GO-Ag1, BC/GO2, and BC/GO-Ag2 dressings were cut to a diameter of 14 mm and sterilized in an autoclave (121 °C for 15 min). The samples were then aseptically placed in a 24-well polystyrene plate, and 1 mL of DMEM culture medium containing 10% (v/v) fetal bovine serum (FBS) and 1% (v/v) penicillin/streptomycin was added to each well. The 24-well plate was incubated at 37 °C and 5% CO_2_ saturation for 24 h. After the incubation period, the supernatant from each well was collected and mixed with 100 μL of DMEM media containing 5 × 10^4^ cells of mouse fibroblasts L-929 per milliliter. The mixture was added to each well, and the plate was incubated at 37 °C, 5% CO_2_, and 95% humidity. Then, the culture medium was removed from the wells and 100 μL of the membrane supernatant samples (BC, BC/GO, and BC/GO-Ag) was added to each well. The plate was again incubated under the same culture conditions for 24 and 48 h. After this period, the liquid media were removed from the wells, and 120 μL of DMEM was supplemented with FBS containing the resazurin reagent (25 mg L^–1^) to measure the mitochondrial activity of the fibroblasts L-929 cells. The plate was then incubated for 4 h under standard culture conditions, and the fluorescence was measured at λ excitation = 560 nm and λ emission = 590 nm using a microplate reader (SpectraMax i3x, Molecular Device, Sunnyvale, USA). Control conditions consisted of cells exposed only to the supplemented DMEM culture medium. The viability of cells in the control group was adjusted to 100% for the calculation of mean values and standard deviation (*n* = 3).

## Results and Discussion

### Morphological, Chemical, and Physicochemical Characteristics of GO1, GO-Ag1, GO2, and GO-Ag2

**Figure 2:**
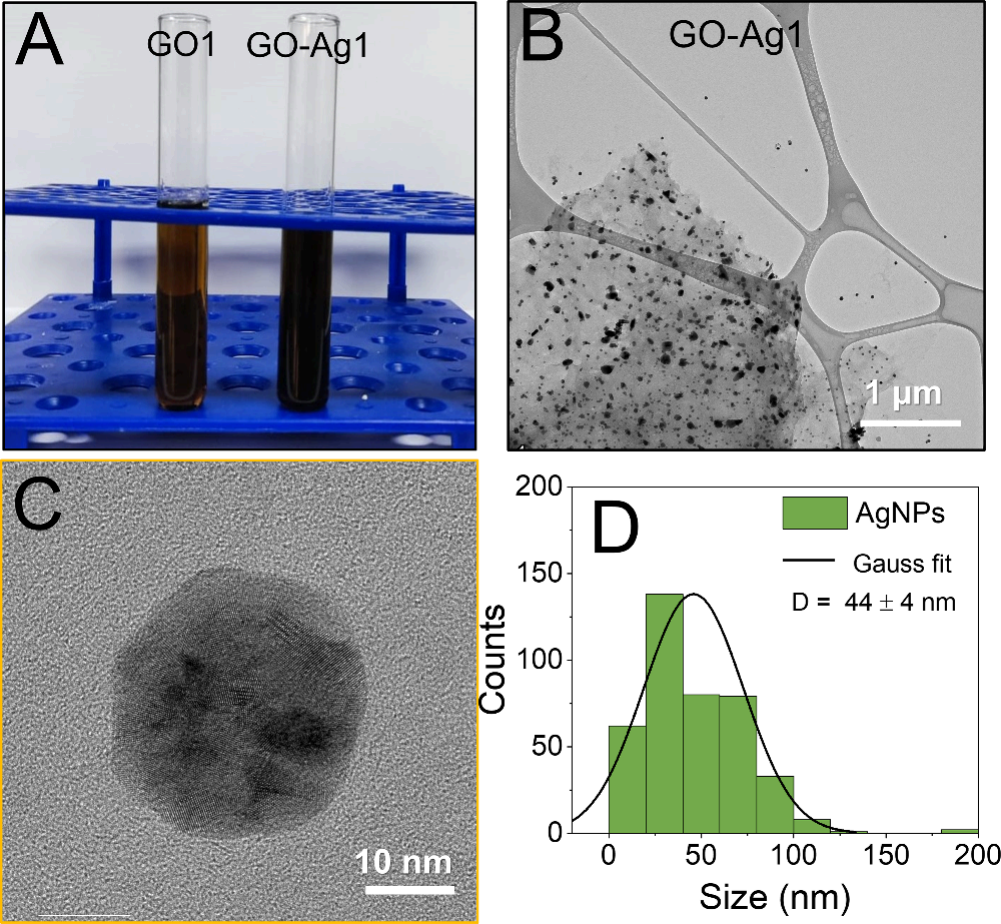
Digital photograph showing the color change of the GO1 dispersion before and after anchoring AgNPs on the surface of the GO sheets (A). TEM images of GO1 sheets decorated with AgNPs, GO-Ag1 (B). Monochromated HRTEM images of the AgNPs attached to the GO sheets (C). Size distribution of the AgNPs on the graphene oxide surface (D).

**Figure 3:**
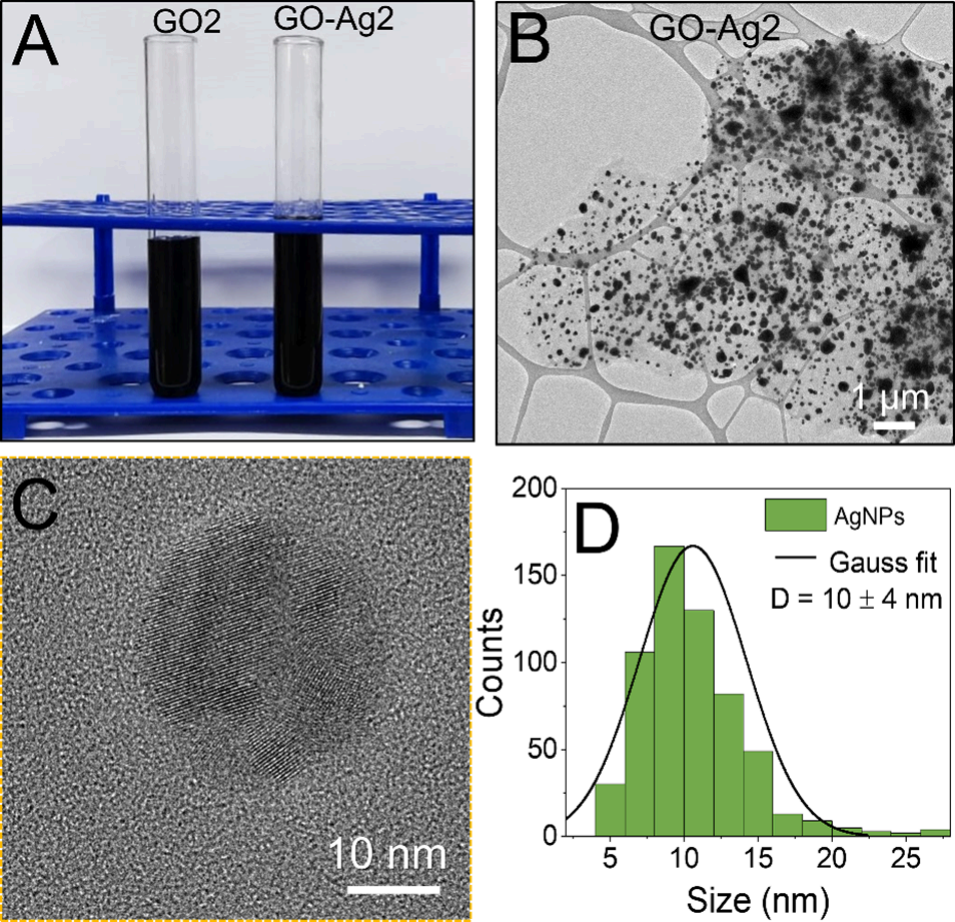
Digital photograph of the GO2 and GO-Ag2 dispersions (A). TEM images of GO2 sheets decorated with AgNPs, GO-Ag2 (B). Monochromated HRTEM image of the AgNPs attached to GO-Ag2 (C). Size distribution of the AgNPs anchored to the graphene oxide surface (D). The size distribution was determined by counting 400 nanoparticles.

The anchoring of silver in graphene sheets by the reduction method causes a color change in the initial sample to the final sample by obtaining GO-Ag1 and GO-Ag2 nanocomposites. The black solution changes to a moss-green color, facilitating the visual identification of the effectiveness of the reaction, as illustrated by the color change in Figures 2A and 3A.

TEM images (Figure 2B,C) revealed that the AgNPs were effectively attached to the GO sheets (Figure 2B). No nanoparticles were found detached from the GO sheets, indicating that the oxygen-containing functional groups on the GO sheets drive the process of nucleation and stabilization, favoring the formation of the particles.^49^ High-magnification TEM images indicate small and spherical-like crystalline particles on the surface of GO-Ag1 (Figure 2C). A polydisperse particle size distribution was observed with most particles less than 50 nm in diameter and an average size of 44 ± 4 nm (Figure 2D). However, particles reaching a size of over 60 nm can be noted (Figure 2B) but do not represent the majority particle size. The size distribution was determined by counting 400 nanoparticles (diameter below 50 nm) and 100 particles (above 50 nm) (Figure S1 and Supporting Information).

GO2 and GO-Ag2 nanoparticles were produced with double concentrations of GO, AgNO_3_, and sodium citrate, as described in the topic of synthesis and characterization of GO-Ag, according to Section 2.3. TEM images for GO-Ag2 (Figure 3B,C) show GO sheets were decorated with AgNPs, which appear as black dots on the surface of the GO sheet. As observed for GO-Ag1, the size distribution graphic (Figure 3D) reveals a polydisperse particle size distribution with quasispherical crystalline structures. However, the particle size distribution shows that the AgNP particles attached to the GO-Ag2 were around 10 nm in diameter (Figure 3D), although populations around 50 nm can also be observed (Figure S2). It is worth mentioning that larger particles can also be visualized in the TEM images (Figure 3B and Figure S2), even though these small agglomerates of AgNPs on the GO-Ag2 surface do not represent the majority of particle size distribution.

**Figure 4:**
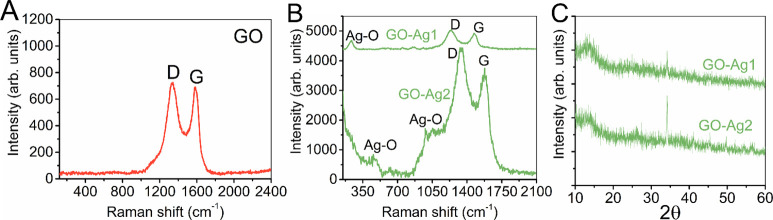
Raman spectra of GO (A), and GO-Ag1 and GO-Ag2 (B). X-ray diffraction patterns of GO-Ag1 and GO-Ag2 (C).

Raman spectroscopy is a highly effective technique for analyzing the crystalline structure of graphene. In the case of graphene, there are two typical bands found in the Raman spectra of graphene: the G band, which refers to the vibration in the plane of sp^2^ carbon atoms, and the D band, which provides valuable insights into the electronic and geometric structure of the material.^50,51^ The high intensity of the D band signal is a characteristic feature of disordered graphene that is often observed in the spectra of the GO samples (Figure 4A). This signal arises due to the chemical oxidation process utilized to exfoliate graphite into GO sheets.^52^ The D and G bands are visible in the Raman spectra of GO (peaks of D at 1342.2 cm^–1^, peaks of G at 1582.23 cm^–1^) (Figure 4A). Similar bands appear in the Raman spectra of GO-Ag1 (D band at 1347.68 cm^–1^, G band at 1590.18 cm^–1^) and GO-Ag2 (D and G bands at 1354.82 and 1577.84 cm^–1^, respectively) (Figure 4B). The peaks at ∼248 cm^–1^^53^ and ∼922 cm^–1^^54^ indicate the Ag–O bond at the spectrum of GO-Ag1 (Figure 4B). Aside from the D and G bands, the GO-Ag2 spectrum displays additional peaks at approximately 459 and 979 cm^–1^, indicating the presence of a Ag–O bond, which is absent in the GO sample.

The X-ray diffractogram (Figure 4C) results in a prominent peak at approximately 13.5°, which is characteristic of GO.^54,55^ In the sample GO-Ag1, the peak at 34.2° refers to the silver anchoring.^56^ This position can be displaced by the degree of oxidation and hydration of the GO sample and by the relative humidity during the X-ray diffraction measurements.^57^ The X-ray diffraction pattern for GO2 shows a peak at 13.6°, while GO-Ag2 exhibits a 34.2° peak, indicating the presence of the Ag–O bond, similar to GO1 and GO-Ag1 (Figure 4C).

The DLS technique was used to determine the particle size distribution for GO and GO-Ag. The hydrodynamic diameters of the GO1 and GO-Ag1 samples were 590.3 and 1301.7 nm, with polydispersity indexes (PDIs) of 0.57 and 0.84, respectively. The hydrodynamic diameters of the GO2 and GO-Ag2 samples were 896.1 and 2144.0 nm with PDIs of 0.590 and 0.894, respectively. The larger hydrodynamic sizes and PDIs for the GO-Ag compared with those of the GO samples are attributed to aggregates likely forming during the process of chemical reduction with citrate. PDI values of up to 0.2 are considered good indicators of stability in water.^58^ A decreased PDI for GO-Ag2 compared to that for GO-Ag1 indicates reduced stability as the concentration of the synthesis reagents increases.

As citrate is added to the reaction media, Ag^+^ ions are concomitantly reduced to AgNPs and GO sheets can also be partially reduced, increasing their tendency for agglomeration. This may explain the higher hydrodynamic values presented for GO-Ag1 and GO-Ag2 compared with that of GO1 and GO2. Although the hydrodynamic diameter of GO-Ag1 increased, the zeta potentials for GO1 and GO-Ag1 suspensions were −33.6 and −36.1 mV, respectively, indicating the abundant presence of oxygen-containing surface groups and good stability in water for both samples.^59^ Zeta potential measurements for GO2 and GO-Ag2 suspensions were 21.3 and 8.85 mV, respectively. Compared to GO-Ag1, GO-Ag2 showed an increased zeta potential, which suggests a decreased stability in water since most of the oxygenated groups were likely participating in Ag–O bonding.

### Physical, Chemical, and Biological Characterization of BC, BC/GO, and BC/GO-Ag Dressings

**Figure 5:**
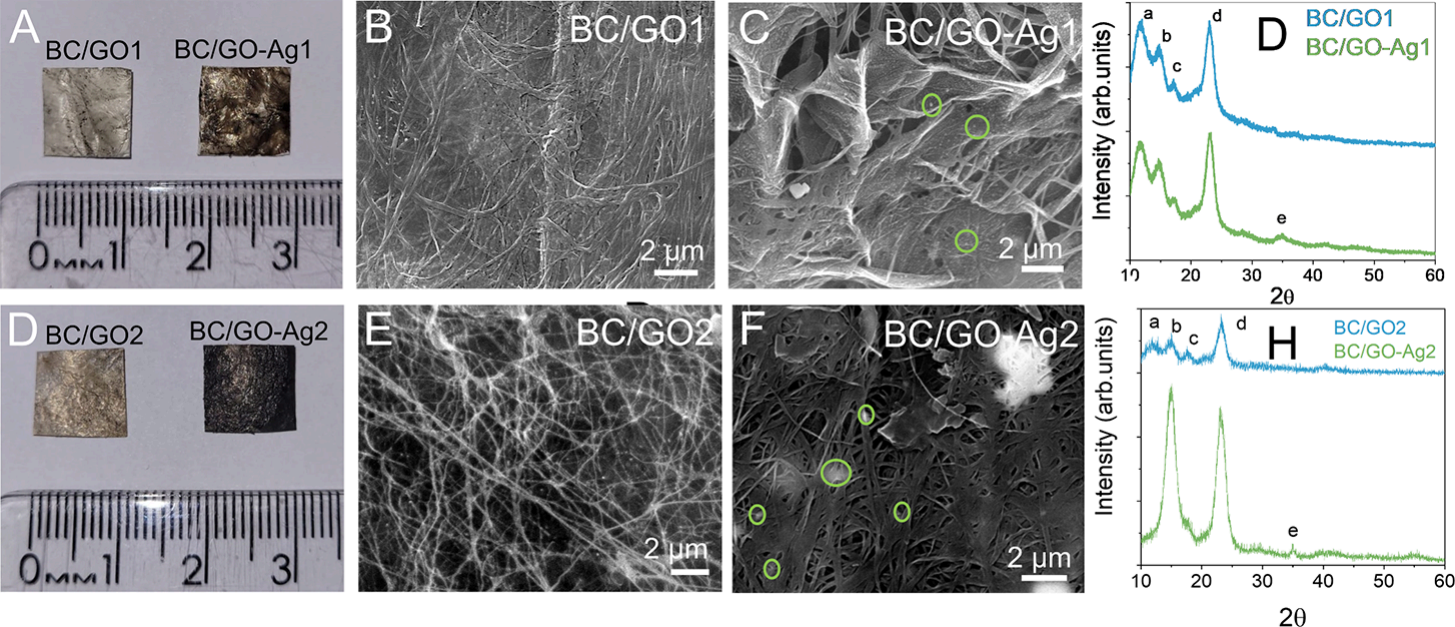
Photographs of BC/GO1 and BC/GO-Ag1 membrane dressings (A). SEM micrographs of BC/GO1 (B) and BC/GO-Ag1 (C). X-ray diffractograms of BC/GO1 and BC/GO-Ag1 (D). Photographs of the BC/GO2 and BC/GO-Ag2 membranes (E). SEM micrographs of the BC/GO2 (F) and BC/GO-Ag2 dressings (G). X-ray diffractograms of the GO2-, BC/GO2-, and BC/GO-Ag2-functionalized dressings (H).

To proceed with the functionalization, the pristine BC membranes were agitated with GO-Ag1 or GO-Ag2 suspensions in a thermostatic bath for 6 h at room temperature. Photos of the resulting BC/GO1 and BC/GO-Ag1 membranes are shown in Figure 5A. SEM micrographs in Figure 5B,C show the polymeric fibers characteristic of the native BC membranes, as described in many previous studies.^41,60^ After impregnation with GO-Ag1, there were no observable differences in the fiber-like structure of the BC membranes (Figure 5B). Therefore, the underlying fiber-like morphology of the BC/GO-Ag1 dressing was found to be similar to that of BC/GO1 (Figure 5B,C), differing only by the presence of the AgNPs, indicated by green circles on the SEM images of the BC/GO-Ag dressings (Figure 5C).

The X-ray diffractograms of BC/GO1 and BC/GO-Ag1 are shown in Figure 5D. The GO1 diffractogram displayed a peak (a) at 11.89°, which is characteristic of GO, as described elsewhere.^61^ In accordance with Gao,^62^ this broad peak position at 11.89° can be easily shifted according to the degree of oxidation and hydration of GO and the relative humidity during the X-ray diffraction analyses. Upon analyses of the diffractograms of BC/GO1 and BC/GO-Ag1 samples, we observe three prominent peaks at approximately 17.1° (b) and 23° (d), characteristic of the presence of type I cellulose, similar to the 16.8 and 22.6° peaks found by Kono et al. for planes (110) and (200)^63^ and an amorphous region assigned to the amorphous cellulose (c) near 18.5°. In addition, BC/GO-Ag1 samples showed a low-intensity peak (e) at 34.7°, which is related to the AgNPs on the GO surface (Figure 5D). In a previous study, a graphene-silver matrix showed a crystalline plane (111) at 38.1°,^64^ slightly different than the peak at 34.7° found herein. Such differences are likely due to the distinct methodologies used to anchor AgNPs to the GO sheets. Pictures of the BC/GO2 and BC/GO-Ag2 membranes are displayed in Figure 5E. SEM images for BC/GO2 and BC/GO-Ag2 dressings show the characteristic nanofibrils of bacterial cellulose and silver aggregates, which are indicated by the green circles in Figure 5G. The X-ray diffractograms of BC/GO2 and BC/GO-Ag2 are shown in Figure 5H. The characteristic peaks of cellulose appear at 17.4 and 23°, referring to the (110) and (200) planes of the polymer were also detected for BC/GO-Ag1 in Figure 5D. Peaks b and c in Figure 5H were covered by peak b of BC/GO-Ag2. The peak characteristic of silver appears at 34.8°, similar to that seen previously in Figure 5D.

**Figure 6:**
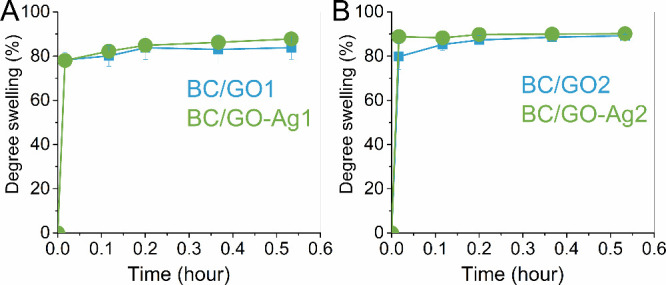
Swelling capacities of the BC/GO1 and BC/GO-Ag1 (A) and BC/GO2 and BC/GO-Ag2 dressings (B) after contacting water for 0.5 h at 25 °C.

The swelling degree (Figure 6A) shows that BC/GO-Ag1 has a swelling profile similar to that of BC/GO1. The same profile is seen for the BC/GO2 and BC/GO-Ag2 dressings (Figure 6B). However, these values were lower than those in the literature^17^ which obtained a 220% swelling degree for native BC. The pristine BC matrix has a hydrophilic nature due to the hydroxyl groups, which quickly interact with water molecules through hydrogen bonding. These hydrogen bonds are likely disrupted after combination with GO, explaining the swelling decrease compared to that of native BC.

The capacity of a wound dressing to absorb exudate is a crucial factor in its effectiveness. For a dressing to work effectively, it should be capable of absorbing as much secretion from the wound as possible. The BC membranes were exposed to a simulated body fluid (SBF) to determine the ability of the wound dressings to absorb physiological fluids. The SBF is a solution of salts dissolved in water that creates osmotic pressure similar to the natural body fluids. The exudate absorption capacity for BC/GO1, BC/GO-Ag1, BC/GO2, and BC/GO-Ag2 dressings are shown in Table 1. The absorption of exudate increased for the samples over the days but with reduced variation between 24 and 72 h. All the samples showed absorption of 87% or more after 24 h. The simulated absorption profile showed a percentage difference of only 6% when comparing BC/GO1 to BC/GO-Ag1 and 1% when comparing BC/GO2 to BC/GO-Ag2 at 72 h.

**Table 1:** Exudate Absorption Capacities Obtained for BC/GO1, BC/GO-Ag1, BC/GO2, and BC/GO-Ag2 Dressings

time (h)	BC/GO1 (%)	BC/GO-Ag1 (%)	BC/GO2 (%)	BC/GO-Ag2 (%)
24	87 ± 3.86	93 ± 2.00	90 ± 2.15	91 ± 7.25
48	88 ± 3.73	94 ± 2.02	93 ± 1.73	94 ± 3.44
72	88 ± 3.75	94 ± 1.73	94 ± 3.73	95 ± 2.91

### Ag Adsorbed and Its Release Profile

Two syntheses were conducted to produce GO-Ag nanocomposites. GO-Ag1 and GO-Ag2 contained 267 and 534 μg mL^–1^ of silver nitrate, respectively. They were then used to functionalize BC to obtain the BC/GO-Ag dressings. After the GO-Ag synthesis, the amount of free silver was measured by using atomic absorption spectroscopy (AAS). The results showed that there were 126.26 and 267.82 μg mL^–1^ of free Ag+ ions released by GO-Ag1 and GO-Ag2, respectively. Based on these results, the remaining amount of silver anchored in the GO sheets is assumed to be 140.74 μg mL^–1^ for GO-Ag1 and 266.18 μg mL^–1^ for GO-Ag2. A different indicator for the silver impregnation result is the percentage of silver adsorption per membrane area, Table 2.

**Table 2:** Adsorption of Ag^+^ Ions on BC/GO-Ag1 and BC/GO-Ag2 Membranes and Their Desorption in Distilled Water after 48 h Exposure

samples	adsorption (μg per cm^–2^ of BC)	desorption (μg per cm^2^ of BC)	desorption total (%)
BC/GO-ag1	38.79 ± 2.05	10.64 ± 0.15	72.56 ± 0.38
BC/GO-ag2	77.93 ± 2.28	29.94 ± 1.37	61.58 ± 1.76

After impregnation with GO-Ag, the obtained BC/GO-Ag dressings can release both adsorbed Ag^+^ ions and silver anchored to graphene (GO-Ag) as antimicrobial agents (Table 2). The release profile of Ag^+^ ions from the modified BC/GO-Ag1 and BC/GO-Ag2 dressings was performed by immersing them in DW for 48 h. The percentage of the release of Ag^+^ ions was 72.56% for BC/GO-Ag1 and 61.58% for BC/GO-Ag2. Although BC/GO-Ag2 has a higher silver release rate, the efficiency of the silver release rate is higher for BC/GO-Ag1 than for BC/GO-Ag2. Silver leaching is related to particle size, the surface area of GO-Ag, and the concentration of GO-Ag deposited on the surface of the BC. In average, GO-Ag1 showed particle size four times larger than GO-Ag2 (FigureS 2D and 3D). Bigger particles dissolve for longer times than for small ones. That is why the concentration of released Ag^+^ is higher for BC/GO-Ag1 than BC/GO-Ag2.

The BC/GO-Ag2 released about 30 μg of Ag^+^ ions per cm^2^ of dressing, a value almost three times higher than shown by BC/GO-Ag1 (Table 2). An improved release of Ag^+^ ions could result in a greater antimicrobial effect. Chook et al.^65^ developed dressings with a similar basic formulation (cellulose and graphene immersed in a solution containing silver and ammonia complexes), showing release values of 78.8, 149.5, and 200 μg of Ag^+^ ions per cm^2^ sufficient to have an antimicrobial activity of between 90.4, 91.3, and 94.4% (10 cm^2^ of membranes) in 4 h of contact against *E. coli.*

### Antibacterial Activity and Cytotoxicity Assays

**Figure 7:**
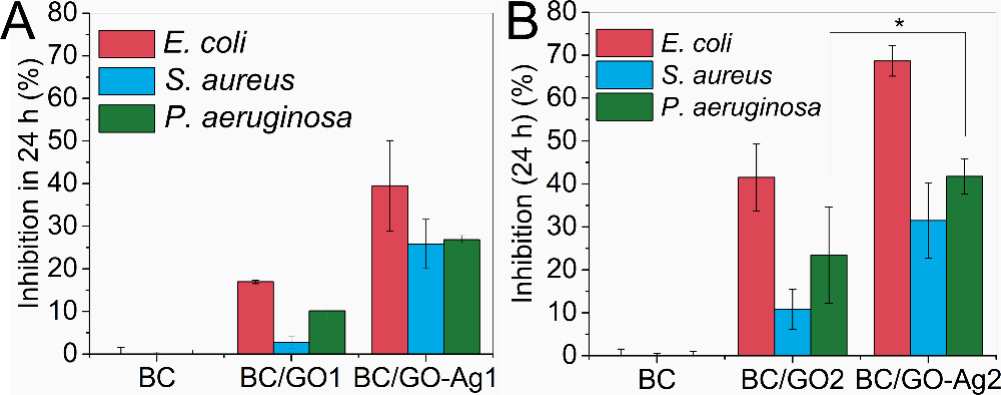
Antibacterial effect of dressings (BC/GO and BC/GO-Ag) obtained from GO-Ag1 (A) and GO-Ag2 (B) at the end of 24 h of incubation against three types of bacteria Gram-positive bacterium *Staphylococcus aureus* and the Gram-negative bacteria *Pseudomonas aeruginosa* and *Escherichia coli*. The ANOVA test and Dunn’s post-test with multiple comparisons were applied. Values with significant differences were considered if *p* < 0.05.

The antibacterial effects of BC/GO1, BC/GO2, BC/GO-Ag1, and BC/GO-Ag2 dressings were evaluated against Gram-positive and Gram-negative bacteria, and the results are shown in Figure 7.

The BC sample did not show antimicrobial activity against any of the bacteria strains investigated (Figure 7A,B). The BC/GO1 and BC/GO2 dressings were more active against *E. coli* followed by *P. aeruginosa*, demonstrating less toxicity to *S. aureus*. The BC/GO1 dressing showed bactericidal activities of 17, 10, and 3%, while BC/GO2 inhibited 41, 23, and 11% of *E. coli*, *P. aeruginosa*, and *S. aureus*, respectively. This finding confirms that GO itself has antibacterial activity and that this effect is relative to the concentration applied, as BC/GO2 with a higher GO concentration showed improved bacterial activity than BC/GO1 (Figure 7A,B). In previous studies, GO showed antibacterial activity against *E. coli* by oxidizing cell membrane components.^66^ This potential was maximized by association with AgNPs, as silver can compromise the structure and composition of the cell wall through ionic interaction.^67,68^

Both BC/GO-Ag1 and BC/GO-Ag2 dressings displayed improved antibacterial properties compared to those of their respective BC/GO1 and BC/GO2. The BC/GO-Ag2 dressing showed an improved bactericidal effect against the three bacterial strains tested compared with BC/GO-Ag1, inhibiting ∼70, 42, and 32% of viable *E. coli*, *P. aeruginosa*, and *S. aureus* cells, respectively. This improved antibacterial property can be attributed to the increased release of Ag^+^ ions per area of the membrane (Table 2). Therefore, impregnation with GO-Ag2 containing higher contents of silver was crucial to impart an improved antibacterial activity to the BC dressings. The commercial dressings Aquacel Ag Extra and Mepilex Ag+ Molnlycke displayed antimicrobial activity against *E. coli*, *P. aeruginosa*, and *S. aureus*, comparable with our BC/GO-Ag2 membranes (Figure S3). Despite Aquacel Ag Extra being labeled with a lower concentration of silver per dressing area, it exhibited better antimicrobial activity compared to Mepilex Ag+ Molnlycke. This indicates that the production process and polymeric matrix impact the release of the active compound.

Our results also suggest that the BC/GO-Ag dressings present two forms of antimicrobial actives in their composition. As revealed in the silver release tests, there is substantial leaching of Ag^+^ ions on the BC surface. The dissolution of the AgNPs attached to GO sheets on the surface of the BC/GO-Ag dressings seems to be responsible for the initial bactericidal effect. As the BC matrix is exposed to fluids over time, isolated particles of GO-Ag can also detach from the surface of the dressings and be dispersed in the suspension, which can become toxic to bacteria. The Ag^+^ ions damage microorganisms by binding to cell proteins with thiol groups (SH). This interaction, especially with proteins and enzymes in the cell membrane, can lead to rupturing of the cell membrane.^49,69^ Because of their small size, AgNPs can also enter cells, causing mitochondrial damage, denaturing ribosomes, interfering with cell proliferation, and inhibiting ATP production.^70^

### Cytotoxicity against Fibroblastic Cells

**Figure 8:**
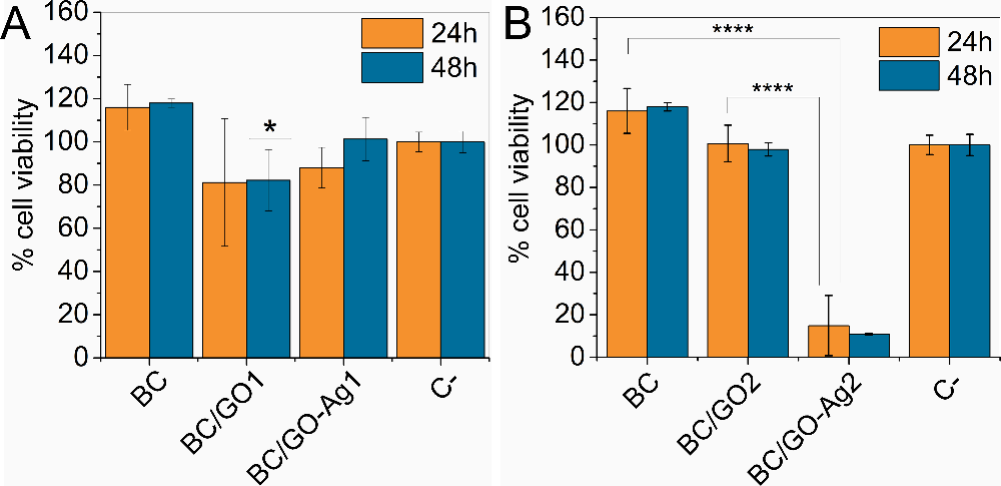
Viability of L-929 fibroblasts after incubation with BC/GO and BC/GO-Ag extracts obtained from synthesis 1 (A) and synthesis 2 (B) at the end of 24 and 48 h of incubation. The ANOVA test and Dunn’s post-test with multiple comparisons were applied. Values with significant differences were considered if *p* < 0.05.

Cell viability was quantitatively evaluated to estimate the biocompatibility of the BC/GO and BC/GO-Ag dressings produced from the two syntheses (1 and 2) by the MTT assay. Figure 8 shows the viability of fibroblastic cells after exposure to liquid media that contacted the dressings for 24 and 48 h. The fibroblast cells show high viability after incubation with extracts from BC/GO and BC/GO-Ag1 (Figure 8A). However, extracts produced from BC/GO-Ag2 were more toxic at both experimental times, 24 and 48 h, showing that higher concentrations of GO-Ag in the dressing composition can negatively affect the biocompatibility of the final product (Figure 8B).

These results suggest that the BC/GO-Ag dressing can be a promising antimicrobial agent, as demonstrated by microbiological tests. However, it is necessary to define a concentration that presents a good bacterial performance but low cytotoxicity. It is crucial to reduce the concentration of silver when producing dressings using synthesis 2. This is because silver is highly toxic to several cells, including fibroblasts, and this can interfere with cell proliferation, tissue regeneration, and wound healing. Further evaluation of the toxicity of BC/GO-Ag1 and BC/GO-Ag2 requires more advanced toxicity studies, such as animal studies, since responses based on cytotoxicity in cell culture are limited. Silver nanoparticles anchored to graphene provide excellent antibacterial properties to BC/GO-Ag2, requiring a significantly lower amount of silver (77.93 μg cm^–2^) compared to other dressings on the market, such as Aquacel Ag Extra (1200 μg cm^–2^)^71^ and Mepilex Ag^+^ Molnlycke (1800 μg cm^–2^).^72^ Therefore, in comparison, the BC/GO-Ag2 membranes demonstrated a strong antibacterial activity with almost 90% less silver than the tested commercial samples. Despite its excellent antibacterial properties, a previous study has shown that Aquacel Ag Extra was toxic to human fibroblasts in culture medium.^73^ Mepilex Ag+ Molnlycke exhibited a highly release of Ag+ ions, resulting in an expressive cytotoxicity against hamster lung fibroblasts.^74^ The cell viability of the commercial dressings was below 40% for different types of fibroblasts, which was similar to the results exhibited by the BC/GO-Ag2 membranes. In this article, our focus is on evaluating the characteristics of BC/GO-Ag1 and BC/GO-Ag2 *in vitro*. Future studies should assess the cytotoxicity *in vivo* to extend the effect of GO-Ag to the different tissues involved in the healing process.

## Conclusions

The materials developed in this study have shown promise as antimicrobial dressings. The design of BC/GO-Ag dressings required much lower concentrations of silver compared to commercial dressings thanks to a nanotechnological process used to obtain the nanocomposites (GO-Ag). The study also highlights the synergistic effect of graphene and silver since the BC membranes modified with GO-Ag presented improved toxicity compared to those modified only with GO. Two different samples of GO-Ag, GO-Ag1 and GO-Ag2, with varying silver contents, were used to impregnate the BC membranes through physical adsorption. The BC/GO-Ag2 dressing released approximately 30 μg of silver per cm^2^ of BC in 48 h, which was enough to inhibit the growth of microorganisms and thereby accelerate the tissue healing process. The BC/GO-Ag2 showed the best antimicrobial action (70%) against Gram-negative bacteria *E. coli* compared to its counterpart BC/GO-Ag1. However, this increased antibacterial activity also resulted in greater toxicity to fibroblast cells. In contrast, BC/GO-Ag1 had lower antibacterial activity but could absorb excess exudate without affecting the proliferation of fibroblast cells. In future studies, it will be essential to adjust the concentration of silver in the GO-Ag nanocomposite to maintain the antimicrobial properties of BC/GO-Ag2 while reducing its toxicity to fibroblast cells. In future studies, complementary biological assays, such as cell migration and wound healing *in vivo*, could be conducted to explore the silver and GO concentrations in the nanocomposite and their toxic effects on living animals.

## Supporting Information Available

TEM micrographs of GO-Ag1 for different regions of GO-Ag1 surface; TEM micrographs of GO-Ag2 surface; antimicrobial activity expressed in terms of percentage of bacterial inhibition of the commercial dressings Aquacel Ag Extra and Mepilex Ag+ Molnlycke (PDF)

## Abbreviations

(BC): bacterial cellulose
(GO): graphene oxide
(GO-Ag): oxide-silver nanoparticles
(AgNPs): silver nanoparticles
(TEM): transmission electron microscope
(HS medium): hydrosulphite of sodium medium
(SBF): simulated body fluid
(DMEM): Dulbecco’s Modified Eagle's Medium
(FBS): fetal bovine serum
