# Supplementary material for: Bacteria-Derived Cellulose Membranes Modified with Graphene Oxide-Silver Nanoparticles for Accelerating Wound Healing
Source: ACS Appl Bio Mater. 2024 Aug 2;7(8):5530–40. doi: 10.1021/acsabm.4c00650 (PMC11337152; doi:10.1021/acsabm.4c00650)
Supplement: Supplementary file 1 — mt4c00650_si_001.pdf [file mt4c00650_si_001.pdf]

Bacteria-Derived Cellulose Membranes Modified with Graphene Oxide-Silver Nanoparticles for Accelerating Wound Healing

Erika Patrícia Chagas Gomes Luz [a], Thamyres Freire da Silva [a], Lidyane Souto Maciel Marques [a], Alexandre Andrade [b], Marcos Vinicius V Lorevice [e], Fabia Andrade [a], Liu Yang [d], Antonio Gomes de Souza Filho [c], Andreia F. Faria [d], Rodrigo Silveira Vieira [a].*

[a] Federal University of Ceará (UFC), Department of Chemical Engineering, 60455-760, Fortaleza, Ceará, Brazil.

[b] Federal University of Ceará (UFC), Department of Pathology and Forensic Medicine, 60430-160, Fortaleza, Ceará, Brazil.

[c] Federal University of Ceará (UFC), Department of Physics, Bloco 922, 60455-760, Fortaleza, Ceará, Brazil.

[d] University of Florida, Department of Environmental Engineering Sciences, University of Florida, 32611-6540, Gainesville, FL, USA.

[e] Brazilian Nanotechnology National Laboratory (LNNano), Brazilian Center for Research in Energy and Materials (CNPEM), 13083-970, Campinas, São Paulo, Brazil.

SUPPORTING INFORMATION

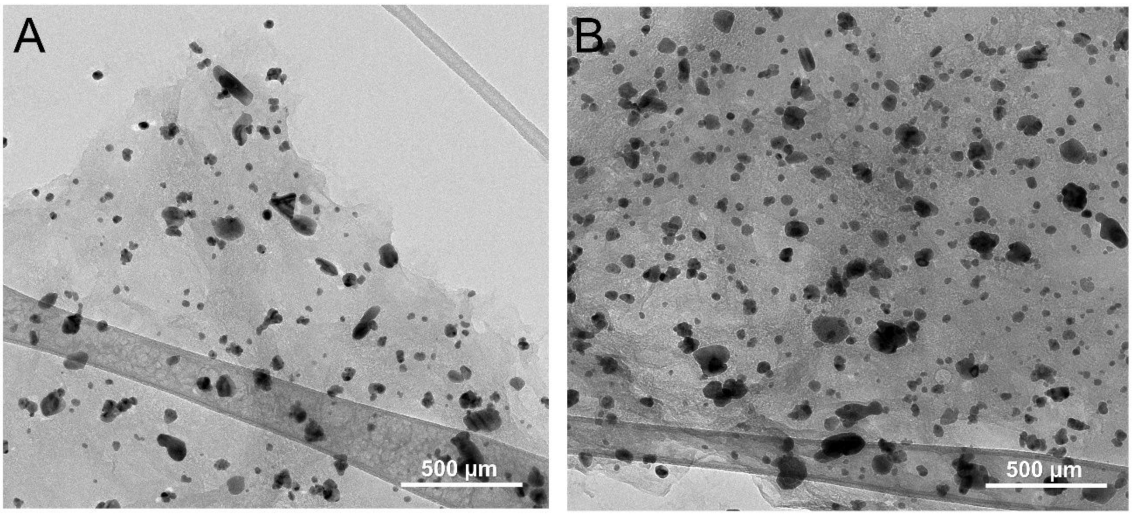

Figure S1: TEM micrographs of GO-Ag1 for one region (A) and for another region (B) of graphene oxide sheets with AgNPs.

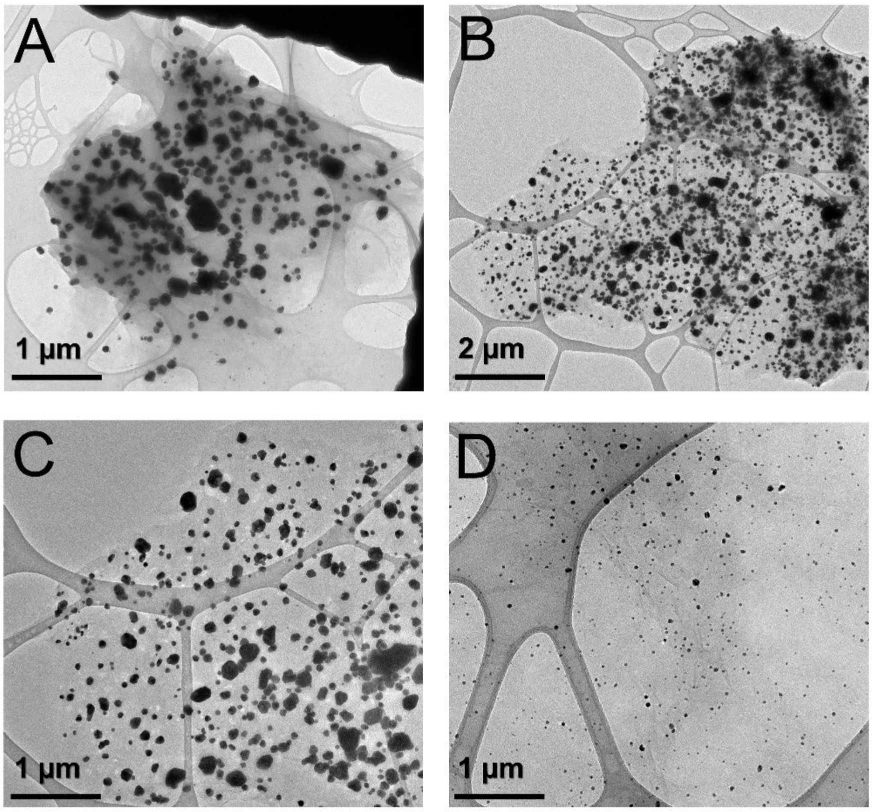

Figure S2: TEM micrographs of GO-Ag2 in deterrents magnifications (A), (B), (C) and (D).

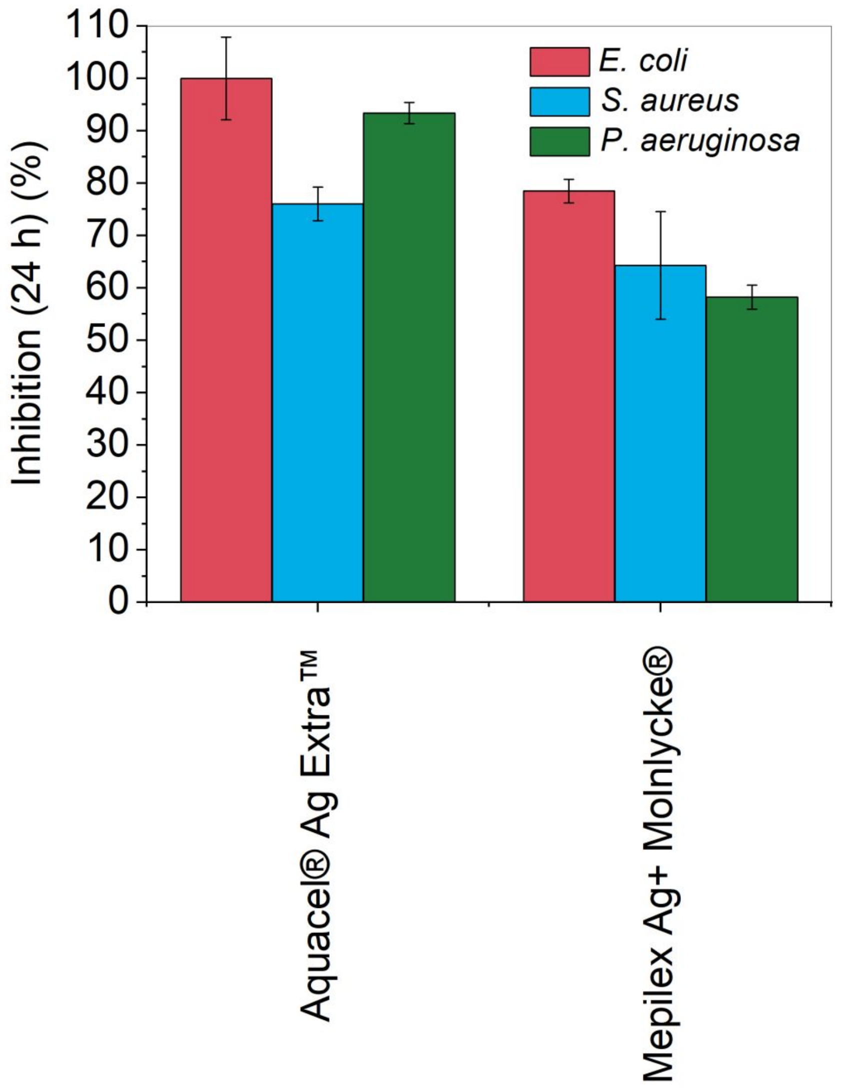

Figure S3: Antimicrobial activity expressed in terms of percentage of bacterial inhibition of the commercial dressings Aquacel® Ag Extra™ and Mepilex Ag+ Molnlycke®.
